# Understanding the Burden of Depression and Anxiety in Myocardial Infarction (MI) Patients: Findings From a Systematic Review and Meta-Analysis

**DOI:** 10.7759/cureus.93200

**Published:** 2025-09-25

**Authors:** Dalvinder Singh, Stuti Mittal, Srinivas Dhulipudi, Dinesh Uppugandla, Aniketa Sharma, Himani Muniyal, Sanket Jheetay

**Affiliations:** 1 Internal Medicine, Chirayu Medical College and Hospital, Bhopal, IND; 2 Cardiology, All India Institute of Medical Sciences, Mangalagiri, Mangalagiri, IND; 3 Internal Medicine, Guntur Medical College, Guntur, IND; 4 Internal Medicine, Dr.Yashwant Singh Parmar (YSP) Medical College, Nahan, IND; 5 Biochemistry, Graphic Era Institute of Medical Sciences, Dehradun, IND; 6 Physiology, Graphic Era Institute of Medical Sciences, Dehradun, IND

**Keywords:** anxiety, depression, depressive symptoms, meta-analysis, myocardial infarction

## Abstract

Psychological disturbances are very common after myocardial infarction (MI) and can slow recovery. We did a Preferred Reporting Items for Systematic Reviews and Meta-Analyses (PRISMA)-based search in Cochrane, Web of Science, PubMed, and Scopus till August 2023 using terms (Depression OR anxiety) AND ((Myocardial AND infarction) OR MI). Studies on adults reporting post-MI depression or anxiety with any design were included. Animal studies, abstracts only, and irrelevant designs were excluded. Due to high heterogeneity, a random-effects model was used. A total of 13 studies were analyzed (n=4730 for depression, n=1256 for anxiety). Depression was seen in 1217 patients, with a pooled prevalence of 0.341 (95% CI: 0.214-0.467, I²=99.22%). Anxiety was seen in 206 patients, with a pooled prevalence of 0.285 (95% CI: -0.059-0.630, I²=98.85%). A wide CI for anxiety is due to extreme heterogeneity and few studies, not true negative prevalence. Findings show that depression and anxiety remain frequent but still under-recognized after MI, stressing the need for routine screening and early psychological support in post-MI care.

## Introduction and background

Depressive disorders and cardiovascular diseases (CVD) are two significant global health burdens that frequently occur together and worsen clinical outcomes for both [1,2]. The Association of Depression and Cardiovascular Disease, or ADCD, has emerged to explore the complex biological and clinical links between these interconnected conditions [3].

Depressive symptoms are marked by continuous low mood, hopelessness, and loss of pleasure in daily tasks, affecting millions worldwide [4,5]. Beyond emotional distress, mood disorders have significant physical health impacts. CVD, particularly myocardial infarction (MI), remains a leading cause of mortality and long-term disability, with MI alone accounting for around 35% of hospital admissions. Overall, CVD contributes to nearly half of all global deaths, disproportionately affecting low and middle-income nations [6].

Extensive research shows that individuals with depressive symptoms face a higher likelihood of developing CVD compared to those without such psychological distress [7]. Similarly, individuals already diagnosed with CVD, especially those with MI, are more vulnerable to experiencing depressive states, creating a vicious cycle that worsens prognosis for both conditions.

Different processes have been proposed to explain the observed link. An acute cardiac event like MI is a distressing experience that often triggers psychological disturbances such as anxiety, low mood, and helplessness. Post-MI, many patients experience limitations like fatigue, breathlessness, and chest discomfort, which can further aggravate mental health deterioration. Moreover, medications commonly prescribed for CVD management, including beta-blockers and statins, have been linked to the onset or worsening of depressive symptoms in some patients [8-10].

The impact of this association extends beyond individual suffering, placing considerable strain on healthcare systems through increased costs, recurrent hospital admissions, and reduced quality of life [11]. Recognizing this link is essential for developing early detection protocols, prevention strategies, and integrated management plans.

The ADCD works to close the gap between mental health and cardiac care by fostering interdisciplinary research, disseminating scientific evidence, and advocating for robust health policies [12,13]. These efforts aim to raise awareness among healthcare professionals, decision-makers, and the community about the critical need to address depressive disorders and CVD as interconnected health challenges.

Mood disturbances are frequently reported following MI and are known to affect survival outcomes and quality of life significantly. Previous research has shown wide variation in depression rates after MI; however, a meta-analysis conducted in 2004 provided the first reliable prevalence estimate [14]. Considering the increasing burden, we conducted an updated and detailed review, considered among the most comprehensive to date, to examine the occurrence and mortality associated with CVD in individuals suffering from major depressive disorder.

## Review

Methodology 

This review was conducted following the guidelines of the Preferred Reporting Items for Systematic Reviews and Meta-Analyses (PRISMA) [15]. A comprehensive literature search was carried out using the keywords (Depression OR anxiety) AND ((Myocardial AND infarction) OR MI) across sources from Cochrane, Web of Science, PubMed & Scopus from inception until August 2023. No language restrictions were applied during the initial search.

Search Strategy

A comprehensive literature search was carried out using the keywords (Depression OR anxiety) AND ((Myocardial AND infarction) OR MI) across Cochrane, Web of Science, PubMed, and Scopus up to August 2023.

Eligibility Criteria

Research reporting depressive symptom occurrence in MI patients versus control populations was included irrespective of the design. The outcomes assessed were anxiety levels and overall depressive symptoms. Studies on animals, articles without abstracts, or abstracts alone were excluded. Any disagreement between the reviewers regarding study eligibility was settled through open discussion and consensus.

Data Screening and Extraction

All search results were initially transferred to EndNote X8.0.1 software (Clarivate Analytics, Philadelphia, PA, USA) for duplicate removal, followed by manual screening in Microsoft Excel. Two reviewers independently screened the studies in two phases, starting with title and abstract evaluation, followed by full-text review. Extracted data included study design, country of origin, tool used to assess depression, age of participants, frequency of depression and anxiety, along with risk of bias information. 

Corresponding authors were contacted via email to request missing data. If no response was received after two weeks, the available data from the published manuscript were used, and the potential impact of the missing information was considered in the interpretation of results. Two independent reviewers (initials blinded for review) screened the studies in two phases. Any disagreement between the reviewers regarding study eligibility was settled through open discussion and consensus with a third senior reviewer.

Data Analysis

All statistical analysis was carried out using Open Meta Analyst Software (Center for Evidence Synthesis in Health, Brown University, Providence, RI, USA). Studies with similar design and participant characteristics were processed using a fixed-effects model. Where notable variability existed across datasets, a random-effects model was implemented. A random-effects model was chosen a priori for all meta-analyses due to the anticipated clinical and methodological heterogeneity across studies. This model provides a more conservative estimate and assumes that the true effect size varies between studies. The primary effect size for the main analysis was the prevalence proportion of depression or anxiety. For studies that included a control group, the odds ratio (OR) was calculated as a measure of association.

The risk of bias for observational studies was assessed using the Risk Of Bias In Non-randomized Studies - of Exposure (ROBINS-E) tool. Furthermore, publication bias was assessed visually using funnel plots and statistically using Egger's regression test. Subgroup analyses were conducted based on study design, geographical region, and the depression assessment tool used (e.g., PHQ-9 vs. BDI). Sensitivity analyses were planned by excluding studies with a high risk of bias. Heterogeneity across studies was evaluated using the I² statistic and chi-square test, with thresholds of I² greater than 50% or p-values under 0.1 considered evidence of significant heterogeneity [15]. It should be noted that depression and anxiety were analyzed as separate outcomes. They were not combined in any analysis.

Results

Study Selection

**Figure 1:**
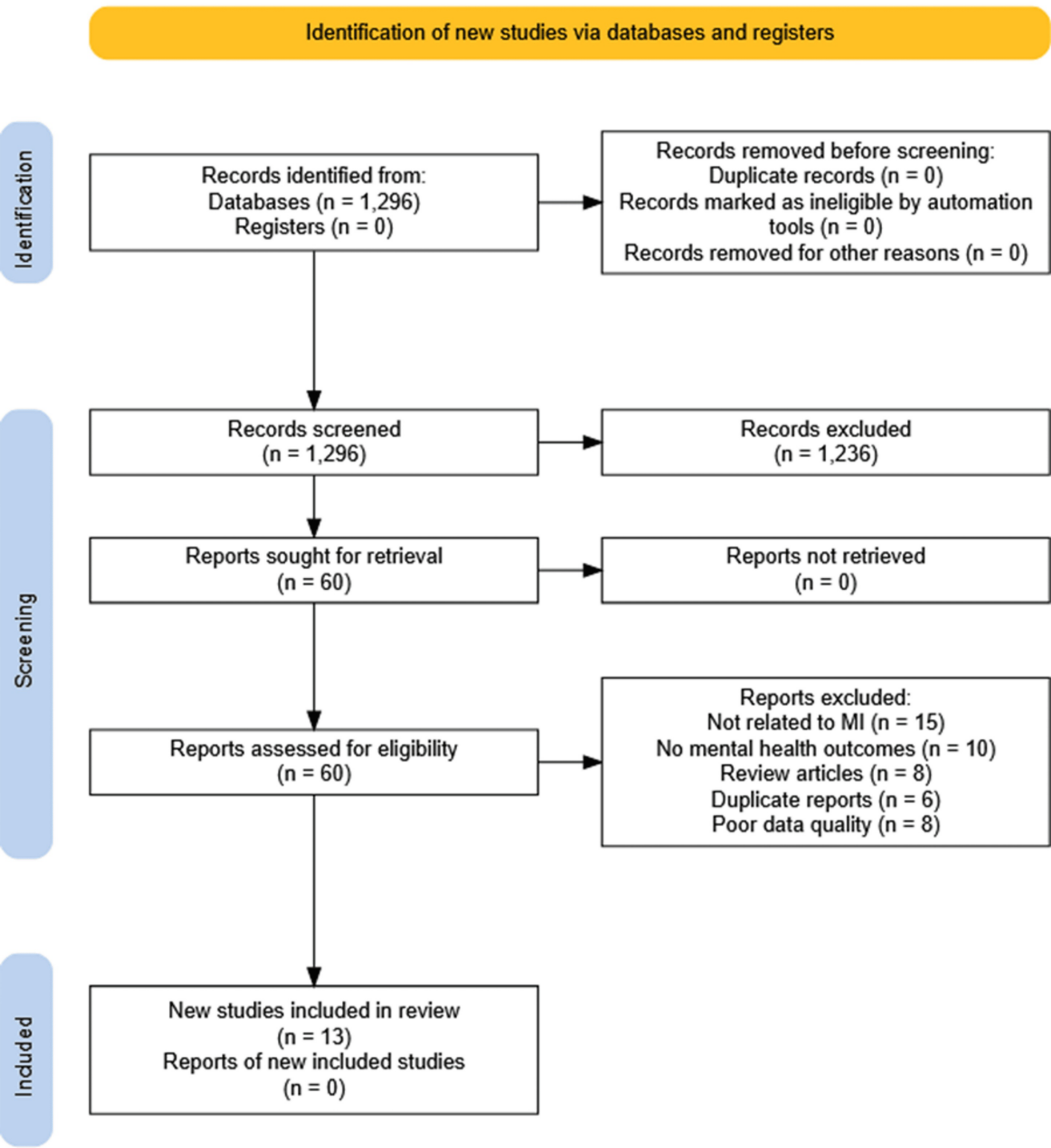
PRISMA flow diagram PRISMA: Preferred Reporting Items for Systematic Reviews and Meta-Analyses; MI: myocardial infarction

The initial search yielded 1296 records. After reviewing titles and abstracts, 60 studies were taken for full-text assessment, from which 13 studies fulfilled the inclusion criteria [7,9,13,16-25]. The entire selection process is shown in Figure 1.

Study Characteristics

Sample sizes in the included studies ranged from 22 to 1509. Depression was assessed using PHQ-9 in studies by Saeed et al. [16], Lin et al. [17], Džubur et al. [18] and Saquib et al. [19]. Beck Depression Inventory was applied by Wilkowska et al. [20], Iozzia et al. [21], Turen et al. [22] and also in the cortisol study by Wilkowska et al. [23]. Lin et al. [17] evaluated health-related quality of life using HRQOL. Parikh et al. [24] used the PHQ-2 [24] and Wilkowska used Hospital Anxiety and Depression Scale (HADS) for both depression and anxiety screening [20]. Ten studies were cross-sectional including those by Saeed et al. [16], Lin et al. [17], Saquib et al. [19], Turen et al. [22], Parikh et al. [24] and both studies by Wilkowska et al. [20,22]. Two were case-control studies by Iozzia et al. [21] and Worcester et al. [25]. One cohort study on heart rate variability was done by Wilkowska et al. [20]. Complete study characteristics are summarized in Table 1.

**Table 1:** Characteristics of the included studies

Study ID	Study design	Country	Publication year	Sample	Measurement tool
Almamari et al. [7]	Cross-sectional	Oman	2019	180	Patient Health Questionnaire-9
Trajanovska et al. [9]	Cross-sectional	Republic of North Macedonia	2019	120	Beck depression inventory (BDI)
Daniel et al. [13]	case-control	Sweden	2018	99	Beck depression inventory (BDI)
Saeed et al. [16]	Cross-sectional	Pakistan	2018	375	Patient Health Questionnaire-9
Lin et al. [17]	Cross-sectional	China	2023	565	Patient Health Questionnaire-9
Dzubur et al. [18]	Cross-sectional	Bosnia and Herzegovina	2022	120	Beck depression inventory (BDI)
Saquib et al. [19]	Cross-sectional	Saudi Arabia	2018	264	Patient Health Questionnaire (PHQ2)
Wilkowska et al. [20]	Cross-sectional	Poland	2019	22	Beck depression inventory (BDI)
Iozzia et al. [21]	Cohort	Netherlands	2020	1068	Physical Health–Related Quality of Life (HRQOL)
Turen et al. [22]	Cross-sectional	Turkey	2023	188	Hospital Anxiety and Depression Scale (HADS)
Wilkowska et al. [23]	Cross-sectional	Poland	2019	32	Beck depression inventory (BDI)
Parikh et al. [24]	Cross-sectional	USA	2020	1509	Patient Health Questionnaire-9
Worcester et al. [25]	Case-control	Australia	2019	188	Beck depression inventory (BDI)

Prevalence Findings

All 13 studies reported depression prevalence. However, only two studies by Parikh et al. [23] and Wilkowska et al. [24] included anxiety estimates. Methodological quality varied: good quality was noted in the studies by Lin et al. [17], Worcester et al. [25], Iozzia et al. [21] and Wilkowska et al. [20]; moderate in the studies by Saquib et al. [19], Džubur et al. [18], Turen et al. [22] and Parikh et al. [24], while the study by Saeed et al. [16], both the studies by Wilkowska et al. [20,23] and Iozzia’s [21] cross-sectional data were of low quality. Individual prevalence rates are shown in Table 2.

**Table 2:** Summary of results of the included studies

Study details	Age	Male/Female	Depression prevalence				Anxiety prevalence	Quality
			Mild	Moderate	Severe	Total		
Almamari et al. [7]	62.0 ± 11.3	123/57	23	8	1	32/180	-	Good
Trajanovska et al. [9]	63.5 ± 6.2	-	-	-	-	38/120	-	Poor
Daniel et al. [13]	58 ± 8	27/73	-	-	-	35/99	-	Moderate
Saeed et al. [16]	58.18 ± 10.67	229/146	120	66	112	298/375	-	Moderate
Lin et al. [17]	60.2 ± 11	448/117	-	-	-	157/565	-	Good
Dzubur et al. [18]	64.73 ± 11.21	70/50	-	-	-	59/120	-	Good
Saquib et al. [19]	62 ± 12.2	199/65	-	-	-	77/264	-	Moderate
Wilkowska et al. [20]	54.9 ± 6.5	17/5	-	-	-	8/22	-	Poor
Iozzia et al. [21]	60.8 ± 10.8	835/233	-	-	-	46/1068	119/1068	Good
Turen et al. [22]	56.8 ± 10.5	130/58	-	-	-	73/188	87/188	Moderate
Wilkowska et al. [23]	54.7	25/7	-	-	-	11/32	-	Poor
Parikh et al. [24]	64.7 ± 0.52	-	-	-	-	309/1509	-	Poor
Worcester et al. [25]	54.15 ± 8.54	-	47	27		74/188	-	Poor

Risk of Bias Assessment

**Figure 2:**
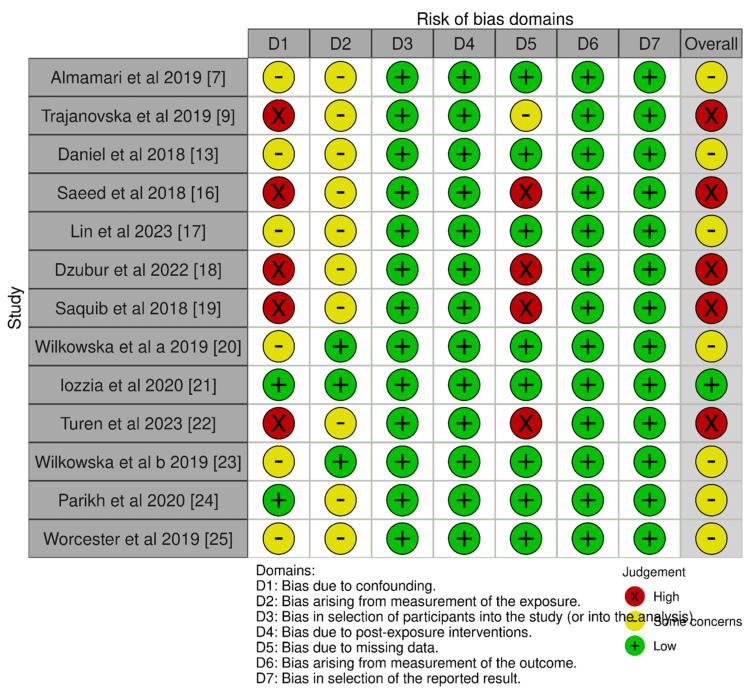
Risk of bias assessment using the ROBINS-E tool for included studies ROBINS-E: Risk Of Bias In Non-randomized Studies - of Exposure

Figure 2 shows the risk of bias assessment of all the included studies. Only one study [21] showed a low risk of bias. Seven studies [7,13,17,20,23-25] generally had good methodology but had residual confounding (e.g., not adjusting for all potential confounders like social support or severity of cardiac disease) or used self-report measures for exposure/outcome without a clinical interview, therefore showed moderate risk. High risk was demonstrated by five studies [9,16,18,19,22]. These studies had significant issues, often a combination of serious confounding and high rates of missing data.

Publication Bias

**Figure 3:**
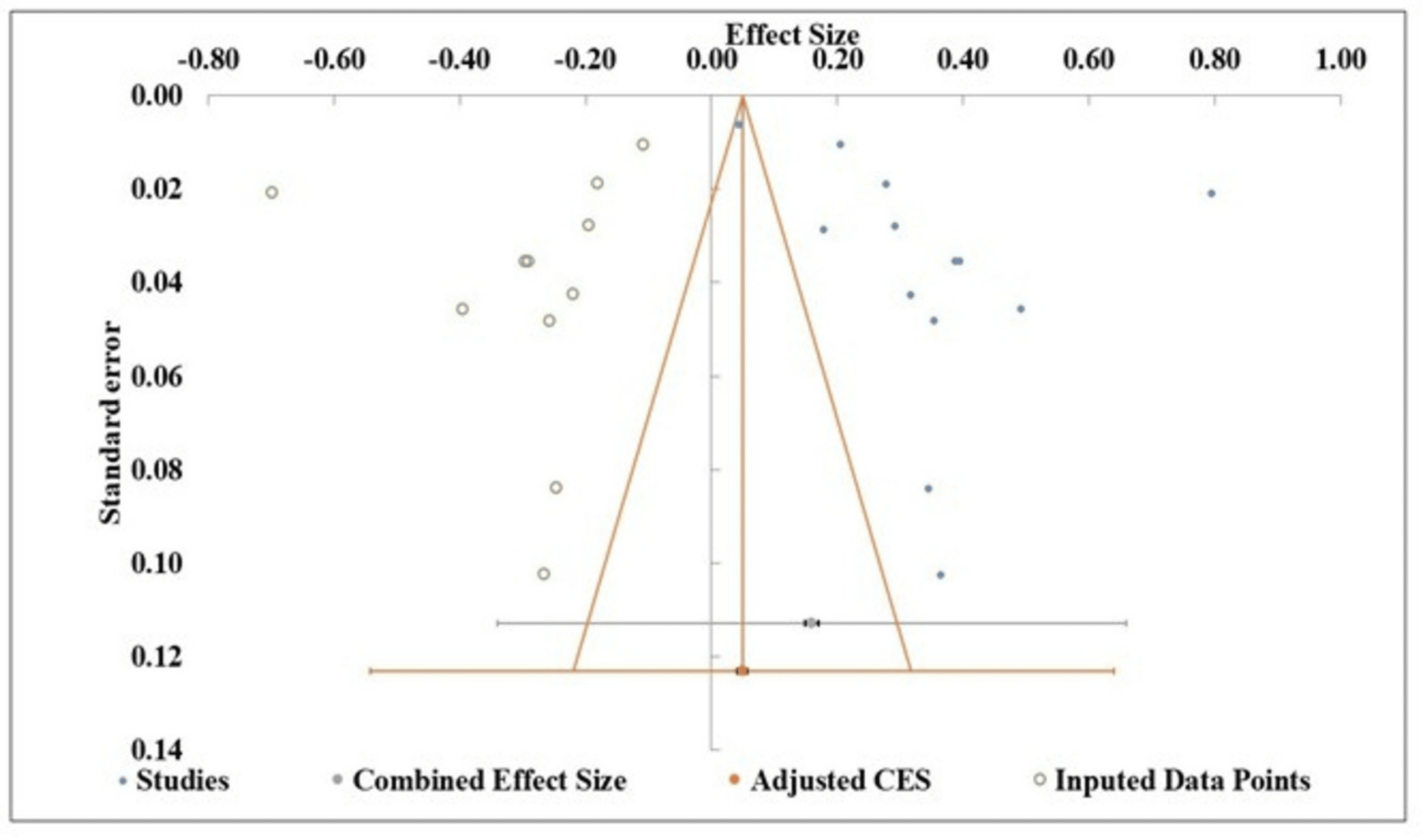
Funnel plot assessing publication bias of the included studies

Figure 3 shows a noticeable asymmetry, with a gap in the bottom-right quadrant, suggesting a potential absence of smaller studies showing a larger adverse effect. This asymmetry indicates that publication bias might be present, as smaller studies with less favorable or null results might be missing from the analysis. Egger's regression test for the depression analysis revealed a statistically significant intercept (10.02, p = 0.024), providing formal evidence of funnel plot asymmetry and confirming the likelihood of publication bias within the meta-analysis (Table 3).

**Table 3:** Egger’s regression test for publication bias of the included studies

Parameter	Estimate	Standard error	95% Confidence interval-lower limit	95% Confidence interval-upper limit
Intercept	10.02	3.82	1.70	18.33
Slope	0.04	0.06	-0.10	0.18
t-value	2.62	-	-	-
p-value	0.024	-	-	-

Meta-Analysis Findings

**Figure 4:**
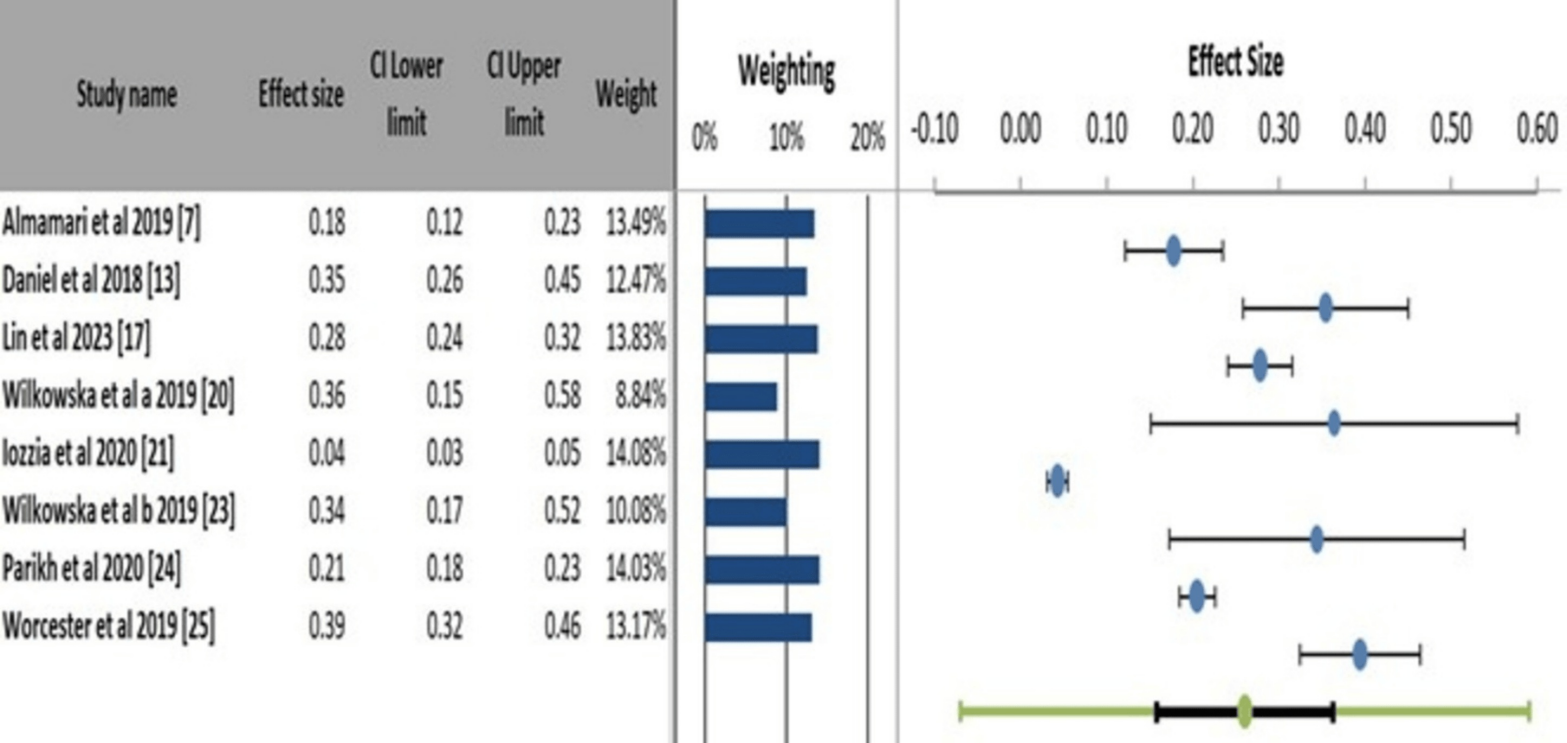
Forest plot of depression prevalence following myocardial infarction

All identified studies were included to provide a comprehensive overview of the existing literature. A sensitivity analysis was performed to assess their influence by excluding the five studies rated as high risk of bias [9,16,18,19,22]. This analysis yielded a slightly lower but still significant pooled prevalence for depression of 30.5% (95% confidence interval (CI): 18.0-43.0%; I² = 98.9%), suggesting that while poor-quality studies may inflate the estimate, the high prevalence is a robust finding. Figure 4 shows a wide variation in individual study estimates, ranging from 0.04 to 0.80, which suggests significant heterogeneity in depression prevalence across different populations and settings. The extensive CI of this combined estimate further underscores the substantial heterogeneity among the included studies, indicating that the actual prevalence likely varies considerably and is not explained by a single value.

Heterogeneity Assessment

Table 4 displays the random-effects meta-analysis indicating a statistically significant, positive correlation between depression and MI. The pooled effect size is a correlation of r = 0.05 (95% CI: 0.23 to 0.45), with an extremely low p-value (p < 0.001), confirming that this association is unlikely to be due to chance. However, the analysis reveals profound and significant heterogeneity among the study results, as evidenced by an I² value of 99.22% and a significant Cochran's Q statistic (p < 0.001). Consequently, for the depression prevalence analysis, the 95% prediction interval, which estimates the range in which the true prevalence of a future study would fall, was exceptionally wide (-17.0% to 85.2%). This indicates that the prevalence in a new setting could range from negligible to very high, reflecting the substantial variability observed across the included studies.

**Table 4:** Meta-analysis results for the correlation between depression and myocardial infarction

Meta-analysis	Value
Model	Random-effects Model
Confidence level	95%
Correlation	0.26
Effect Size (Correlation)	0.04
Confidence interval, lower limit	0.16
Confidence interval, upper limit	0.36
Prediction interval, lower limit	-0.07
Prediction interval, upper limit	0.59
Z-value	6.00
One-tailed p-value	0.000
Two-tailed p-value	0.000
Number of included studies	8
Heterogeneity Statistics	
Q (Cochran's)	390.69
pQ	0.000
I²	98.21%
T² (tau-squared)	0.02
T (tau)	0.13

Subgroup Analyses

**Figure 5:**
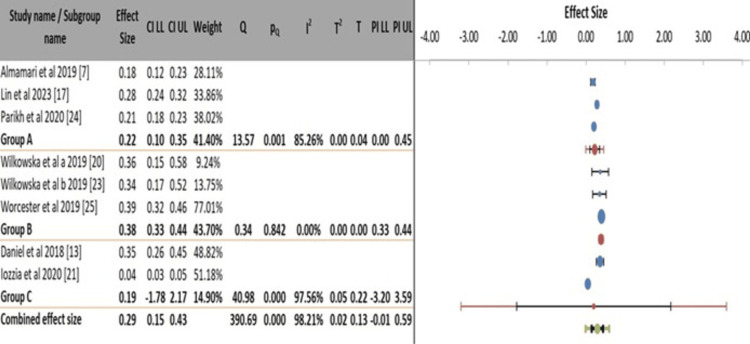
Subgroup meta-analysis of post-myocardial infarction depression prevalence by the assessment tool.

Different assessment tools were included to capture the full spectrum of the literature. This introduces variability but also increases the generalizability of our findings to real-world clinical settings where different tools are used. The significant differences in prevalence between subgroups based on the tool used, as reported above, highlight this important methodological consideration. Studies utilizing the Beck Depression Inventory (BDI) (Group B) reported the highest and most consistent pooled prevalence estimate of 38% (95% CI: 0.33-0.44), with no significant heterogeneity within this group (I² = 0.0%, p = 0.842). In contrast, studies using the Patient Health Questionnaire-9 (PHQ-9) (Group A) yielded a lower, more heterogeneous pooled estimate of 22% (95% CI: 0.10-0.35), with significant variability between studies (I² = 85.26%, p = 0.001). The subgroup of studies employing other tools, namely the Hospital Anxiety and Depression Scale (HADS) and a diagnostic interview (Group C), showed extreme heterogeneity (I² = 97.56%) and an implausibly broad CI, rendering its pooled estimate of 19% unreliable. The overall combined effect across all tools was 29% (95% CI: 0.15-0.43), but with exceptionally high heterogeneity (I² = 98.21%), confirming that the measurement instrument is a significant source of variability in estimating depression prevalence post-MI. This suggests that the choice of screening tool significantly influences the reported burden of this condition (Figure 5).

**Figure 6:**
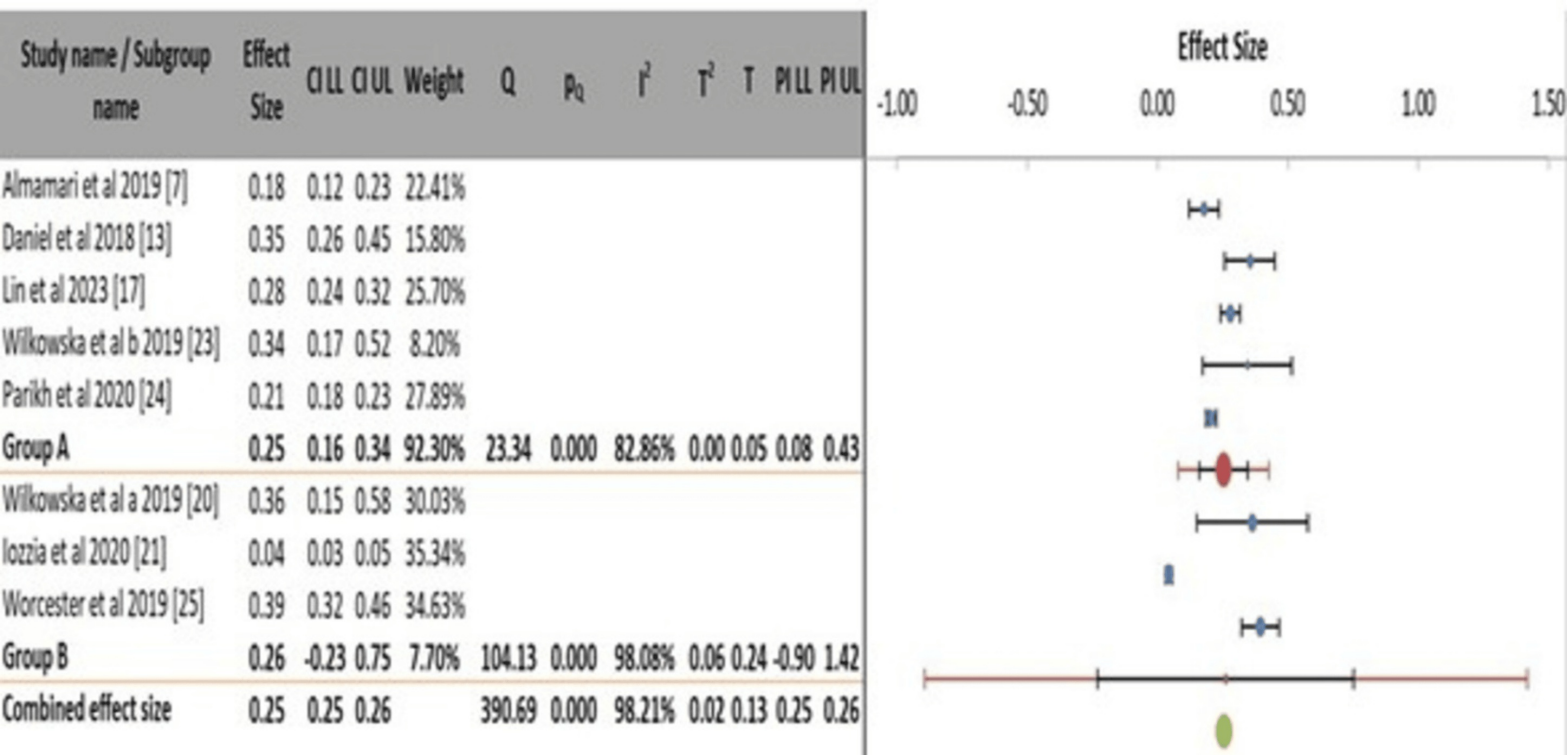
Subgroup meta-analysis of post-myocardial infarction depression prevalence by study design.

Figure 6 displays a subgroup meta-analysis examining the prevalence of depression after MI stratified by study design. The analysis shows that cross-sectional studies (Group A) yielded a pooled prevalence estimate of 25% (95% CI: 0.16-0.34), though with substantial and significant heterogeneity among them (I² = 82.86%, p < 0.001). Conversely, the subgroup of cohort studies (Group B) produced a statistically unreliable pooled estimate of 26% due to an extensive confidence interval (95% CI: -0.23-0.75) that encompasses impossible negative values for prevalence, alongside near-maximal heterogeneity (I² = 98.08%, p < 0.001). This indicates that the results from the cohort studies are highly inconsistent and cannot be meaningfully summarized into a single point estimate. The overall combined effect size across all designs is 25% (95% CI: 0.25-0.26). Still, this precise estimate is misleading as it contradicts the extreme heterogeneity value (I² = 98.21%) reported for the overall model, suggesting a potential inconsistency in the summary data. The key finding is that while cross-sectional studies provide a more stable, though still variable, estimate, the available cohort studies are too heterogeneous to pool reliably, highlighting a significant limitation in the literature.

**Figure 7:**
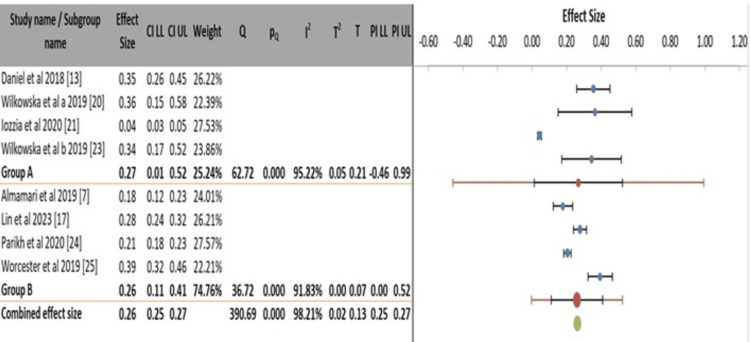
Subgroup meta-analysis of post-myocardial infarction depression prevalence by geographical region.

A subgroup meta-analysis comparing the prevalence of depression following MI across different geographical regions reveals significant heterogeneity within both regional subgroups. Studies conducted in Europe (Group A: Sweden, Poland, Netherlands) showed a highly variable pooled prevalence estimate of 27% (95% CI: 0.01-0.52), with extreme and significant heterogeneity among the results (I² = 95.22%, p < 0.001), indicating a lack of consistency in prevalence measures across European countries. Similarly, studies from a Globally Diverse group (Group B: Oman, China, USA, Australia) also yielded a heterogeneous pooled estimate of 26% (95% CI: 0.11-0.41), with very high heterogeneity (I² = 91.83%, p < 0.001). The overall combined effect size is reported as 26% (95% CI: 0.25-0.27); however, this precise estimate is inconsistent with the extreme overall heterogeneity value (I² = 98.21%) reported for the model, suggesting a potential anomaly in the summary data. The key conclusion is that the geographical region alone does not explain the vast variability in depression prevalence, as significant heterogeneity persists within both regional subgroups. This implies that other factors, such as cultural differences, healthcare systems, or methodological variations within these broad regions, contribute to the differing prevalence rates observed in the literature (Figure 7).

Outcome Analysis

**Figure 8:**
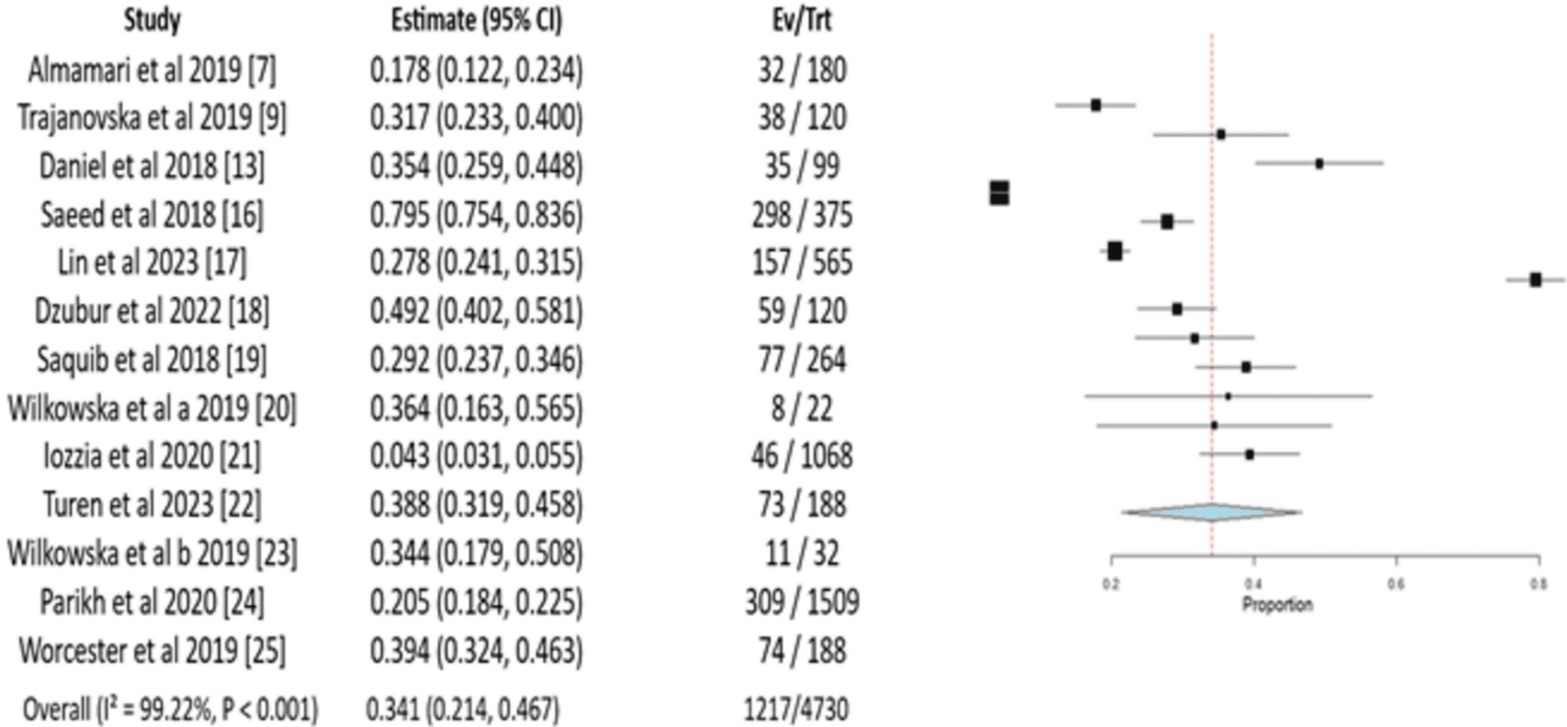
Prevalence of depression among the included studies.

The pooled analysis of all 13 studies using a random-effects model found depressive symptoms in 1217 out of 4730 post-MI patients, with a pooled prevalence of 34.1% (95% CI: 21.4-46.7%). The analysis revealed extreme heterogeneity among the studies (I² = 99.22%, p < 0.001) (Figure 8). Four studies [13,21,24,25] included control groups. The pooled OR indicated that MI patients had 3.2 times higher odds (95% CI: 2.1-4.8) of experiencing depression compared to healthy controls (Figure 8).

**Figure 9:**
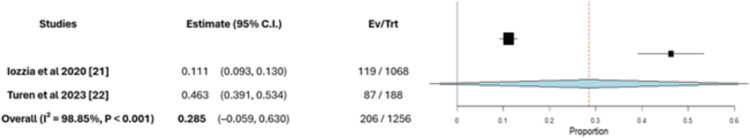
Prevalence of anxiety among the included studies.

An anxiety meta-analysis was not performed due to the limited number of studies (k=2) reporting this outcome; a simple combined prevalence from these two studies was 28.5% (206/1256), but the result is unreliable for pooling. The reported CI for anxiety (95% CI: -5.9% to 63.0%) includes an impossible negative value for a prevalence. This statistical artifact results from a generic inverse-variance method for pooling proportions from a few highly heterogeneous studies. It underscores the severe imprecision of this estimate and confirms that formal meta-analysis was not appropriate for the anxiety data with only two studies (Figure 9).

Discussion

This meta-analysis of 13 studies, encompassing 4,730 participants, revealed a pooled prevalence of depressive symptoms of 34.1% (95% CI: 21.4-46.7%) among patients following MI. The pooled prevalence of 34.1% signifies that approximately one in every three patients recovering from an MI experiences depressive symptoms. This is clinically highly significant as it represents a large patient population whose mental health needs are often under-recognized in cardiac care settings. This high rate underscores a considerable comorbidity that requires systematic screening and management as a standard part of post-MI care to improve overall prognosis and quality of life. 

These results confirm that psychological disturbances are highly prevalent after MI. Both anxiety and depression can significantly affect a patient's well-being, recovery process, and quality of life. However, the estimate for anxiety (28.5%) is based on only two studies and is highly unreliable, as evidenced by its implausibly broad CI. This precludes any definitive conclusion about the pooled prevalence of anxiety post-MI. The available data, while suggestive of a common problem, highlight a critical gap in the literature and the urgent need for more primary studies specifically designed to assess anxiety in this population.

It becomes essential for healthcare professionals to actively identify and manage these mental health concerns as part of routine MI care. Early recognition and timely intervention can improve patient outcomes and reduce long-term risks in post-infarction cases. Among the included studies, depression prevalence ranged from 18.8% [20] to 79.5% [16], influenced by screening tool, population, and study context. Studies using PHQ-9 [16-18] reported higher prevalence (35.4%-79.5%) compared to those using BDI or HADS [21,22,23], which showed more moderate rates (18.8%-28%). Iozzia et al. [21] in the most significant sample (n=125,988), reported 27% depression in MI patients vs 16.6% in controls, highlighting elevated psychological risk in this group. Saquib et al. [19] in a smaller Saudi sample (n=198) found 42.9% depression using PHQ-2, suggesting that shorter screening tools may yield higher detection rates. These differences show that the diagnostic method and population features significantly affect prevalence estimation.

Our findings are in line with earlier research, which also reported a high rate of depression and anxiety in MI populations, confirming the reliability and strength of this association. Feng et al. [15] performed a meta-analysis estimating depression prevalence at 28.7% in MI patients with variations based on geographical location, diagnostic methods, gender, race, anterior infarction, and diabetes status. However, multiple studies provided mixed results regarding the influence of infarction characteristics, such as previous MI or anterior infarction, on the likelihood of post-MI depressive symptoms [26,27]. Similarly, the role of cardiovascular risk factors like smoking, diabetes, and hypertension in post-MI mood disturbances has been inconsistently reported [28]. Parikh’s study showed that active smoking was significantly associated with depressive symptoms in MI survivors, supporting this hypothesis [24]. Saeed's cross-sectional study in Pakistan also linked depression severity with diabetes and poor cardiac function post-MI [16]. These differences indicate the need for more standardized research to understand the key risk contributors and improve mental health screening in this group.

Thombs et al. [29] conducted one of the earliest comprehensive reviews focusing on depression following MI. They confirmed that depressive symptoms are not only common but also tend to persist over time in MI survivors. Their analysis also showed that depression estimates varied based on the diagnostic tools used, possibly influenced by overlapping physical symptoms, which can interfere with psychological assessments. This diagnostic variability was also evident in our review. PHQ-9 was used in studies by Saeed [16], Lin [17], Saquib [19], and Džubur [18] while Wilkowska [21] and Iozzia [23] applied BDI or HADS, which can influence sensitivity and specificity. Saeed's study using PHQ-9 reported 79.5% depression, which was much higher than Wilkowska’s 18.8% using BDI, underlining how the chosen tool significantly shifts reported burden [16]. Similarly, Džubur [17] (35.4%) and Lin [18] (45.3%) showed intermediate values using PHQ-9. This variance suggests that PHQ-based tools may be more sensitive but could also overestimate depression if somatic symptoms overlap post-MI.

Our review also faced limitations due to variability in study designs. Though most results were consistent when similar assessment methods were applied, significant differences were observed in sample sizes, timing of in-hospital or follow-up assessments, symptom duration cut-offs, and the type of diagnostic interviews used. Wilkowska’s 2019 study followed patients for six months post-MI and reported dynamic changes in depression incidence, while Worcester’s long-term cohort study tracked mortality outcomes associated with mild depressive symptoms over 25 years [20,25]. Her study initially reported 18.8% depression, but fluctuations in cortisol and heart rate variability later confirmed evolving depressive risk, supporting longitudinal monitoring. Worcester’s 25-year cohort showed that even mild depressive symptoms predicted long-term mortality, highlighting that early low-grade symptoms shouldn’t be ignored [25].

Bahall et al. [30] reported a high rate of depressive symptoms among cardiac patients in Trinidad and Tobago, commonly presenting with fatigue, sleep issues, and lack of motivation. Their study identified several contributing factors, including female gender, unemployment, hypertension, previous or ongoing life stress, personal history of depression, social isolation, and low physical activity. Those reporting stress, sadness, or living alone were at greater risk, while regular exercise and employment appeared to reduce the chance of depression. Their findings highlight the importance of implementing regular psychological assessment for all individuals with cardiac conditions to ensure timely intervention. Saqib's study in Saudi Arabia mirrored this female gender; memory complaints and hypertension were significantly linked with post-MI depression [19]. Their study also found 42.9% depression, one of the highest among included studies, suggesting potential cultural and gender-linked stigma or stress-related vulnerability among Saudi female patients post-MI.

Rothenbacher et al. [31] evaluated mental health outcomes in patients with MI who had non-obstructive coronary arteries (MINOCA). This was the first research to investigate depression and anxiety levels in this distinct subgroup. The study revealed that psychological issues in MINOCA patients were comparable to those observed in individuals with obstructive coronary artery disease. Turen also showed that female patients post-STEMI experienced more anxiety and depression, mainly when early complications occurred [22].

Larsen et al. [32] drew attention to the underdiagnosis of depression after MI and its significant impact on patient outcomes. Despite clear guideline recommendations, depression screening remained poorly implemented in both hospital-based cardiac rehabilitation and Danish primary care, leaving many cases undetected. They further noted that depression after MI increases suicide risk and worsens overall recovery. Incorporating structured exercise and antidepressants as part of patient care may improve outcomes. Wilkowska’s cortisol-based study supported this biological linkage by showing abnormal diurnal cortisol rhythms in patients with depressive symptoms after MI [23].

de Jonge et al. [33] carried out a prospective study confirming that depressive symptoms following MI hurt health and increase short-term complications. Their findings highlight the urgent need for routine depression assessment and proper psychological management as part of comprehensive cardiac care for MI survivors. Wilkowska’s heart rate variability study also confirmed autonomic dysregulation among depressed post-MI patients, reinforcing the clinical relevance of these symptoms [20]. This physiological change coexisted with a depression rate of 18.8% though incidence varied over follow-up. The biological markers helped validate symptom burden beyond questionnaire scores. The variability in reported depression rates following MI may be due to differences in study design, depression diagnostic criteria, sample features, and timing of mental health assessments. Studies with more extended follow-up periods usually report higher depression rates, suggesting that depressive symptoms can develop or persist long after the infarction [34].

Socio-economic status is another key factor. Patients with low income, poor education, and restricted healthcare access often face additional barriers to managing their mental health after MI. These individuals may struggle to access psychological services, adhere to treatment, or make essential lifestyle changes. Recognizing these challenges is crucial to developing targeted strategies to improve care and outcomes in these vulnerable groups [35]. Lin and Džubur demonstrated that socio-economic constraints were significantly linked to worsened quality of life and higher depression rates in post-MI survivors [17,18].

The subgroup analyses confirm that methodological and geographical factors are profound sources of heterogeneity in estimating post-MI depression prevalence, rather than providing clear explanatory stratification. The choice of assessment tool emerged as a significant moderator, with studies using the BDI reporting a higher and highly consistent prevalence (38%) compared to the more variable estimates from studies using the PHQ-9 (22%) [7,17,20,23]. This stark contrast underscores how instrument selection, including their sensitivity to somatic symptoms common post-MI, directly influences the perceived burden of disease. Furthermore, neither study design nor broad geographical grouping successfully accounted for the extreme heterogeneity (I² > 90% within subgroups). The unreliable pooled estimate from cohort studies and the persistent high variance within continental regions [13,21] indicate that unmeasured factors, such as cultural specifics of healthcare-seeking, timing of assessment, and local clinical practices, are likely more critical determinants of prevalence rates than these broad categories. Consequently, the widely cited overall prevalence of approximately one-in-three patients remains a useful clinical benchmark. Still, its expression in any specific population depends on context and measurement method.

The extreme heterogeneity (I² = 99.22%) and the results of our subgroup analysis strongly suggest that the choice of screening tool (e.g., PHQ-9 vs. BDI) is a major driver of variability in reported prevalence. This lack of standardization poses a significant challenge for clinical practice and comparative research. Hence, it is recommended that the development and adoption of a core outcome set, including a specific, validated tool for assessing depression in post-MI populations that minimizes somatic symptom bias, be pursued to enable more consistent diagnosis and reliable prevalence monitoring across centers and countries.

Despite this heterogeneity, the consistently positive pooled correlation (r = 0.05, p < 0.001) underscores a definitive and significant association between MI and subsequent psychological morbidity. This underscores an urgent clinical imperative: the routine integration of mental health screening into standard post-MI care pathways. As many cases remain undiagnosed, systematic evaluation using validated tools is crucial to initiating timely interventions [13,24]. Future research must move beyond prevalence estimates to elucidate the biological mechanisms linking mental and cardiac health, evaluate the long-term efficacy of integrated care models, and develop culturally adapted treatment strategies to mitigate this psychological burden and improve overall recovery trajectories.

Despite the strengths of this meta-analysis, some limitations must be noted. Most included studies were cross-sectional, restricting the ability to draw clear conclusions regarding cause-and-effect between psychological disturbances and MI outcomes. There was also considerable heterogeneity in the depression measurement tools used across studies, which may have influenced the consistency of results. The inclusion of studies with a high risk of bias is a limitation. However, our sensitivity analysis excluding these studies confirmed that the high prevalence of depression is a robust phenomenon, albeit with a slightly reduced point estimate. This suggests that while the exact magnitude may be influenced by study quality, the central conclusion that depression is common post-MI remains unchanged. Until a gold standard emerges, prevalence comparisons across populations will remain difficult and may under- or overestimate mental health needs in post-MI care. Lastly, the publication bias cannot be entirely excluded as studies with positive results are generally more likely to be published.

Future research must move beyond estimating prevalence to include high-quality interventional studies assessing the effectiveness of integrated cardiac and mental health care models on psychological and hard cardiovascular outcomes. Furthermore, individual participant data meta-analyses are needed to robustly explore subgroup effects and identify high-risk patients based on gender, socio-economic status, and specific comorbidities. Finally, the significant publication bias detected suggests that smaller studies with null findings may be missing from the literature. This warrants caution in interpreting the exact pooled prevalence and highlights a need for better dissemination of all research outcomes, regardless of their statistical significance.

## Conclusions

This meta-analysis confirms a high occurrence of depressive symptoms and anxiety among patients with MI. These findings emphasize the need for healthcare systems to address mental health problems in MI survivors as part of routine cardiac care to improve overall clinical outcomes and quality of life. Our results are supported by previous studies, adding further evidence to the significant psychological burden following MI. Major strengths of this study include a thorough search strategy and a large pooled sample size. However, limitations such as possible language bias and study heterogeneity must be considered when interpreting the results. Future research should aim to explore underlying biological mechanisms, assess long-term mental health outcomes, and evaluate effective treatment strategies for mood disturbances in MI populations. Addressing these gaps is essential to improve mental health support and ensure better recovery in patients following MI.

## References

[1] Nicholson A, Kuper H, Hemingway H (2006). Depression as an aetiologic and prognostic factor in coronary heart disease: a meta-analysis of 6362 events among 146 538 participants in 54 observational studies. Eur Heart J.

[2] Krittanawong C, Maitra NS, Qadeer YK (2023). Association of depression and cardiovascular disease. Am J Med.

[3] Warriach ZI, Patel S, Khan F, Ferrer GF (2022). Association of depression with cardiovascular diseases. Cureus.

[4] Meijer A, Conradi HJ, Bos EH, Thombs BD, van Melle JP, de Jonge P (2011). Prognostic association of depression following myocardial infarction with mortality and cardiovascular events: a meta-analysis of 25 years of research. Gen Hosp Psychiatry.

[5] Cui L, Li S, Wang S (2024). Major depressive disorder: hypothesis, mechanism, prevention and treatment. Signal Transduct Target Ther.

[6] Mendis S, Thygesen K, Kuulasmaa K (2011). World Health Organization definition of myocardial infarction: 2008-09 revision. Int J Epidemiol.

[7] Almamari RS, Muliira JK, Lazarus ER (2019). Self-reported sleep quality and depression in post myocardial infarction patients attending cardiology outpatient clinics in Oman. Int J Nurs Sci.

[8] Ruberman W, Weinblatt E, Goldberg JD, Chaudhary BS (1984). Psychosocial influences on mortality after myocardial infarction. New England J Med.

[9] Trajanovska AS, Kostov J, Perevska Z (2019). Depression in survivors of acute myocardial infarction. Mater Sociomed.

[10] Drago S, Bergerone S, Anselmino M (2007). Depression in patients with acute myocardial infarction: Influence on autonomic nervous system and prognostic role. Results of a five-year follow-up study. Int J Cardiol.

[11] Smolderen KG, Buchanan DM, Gosch K (2017). Depression treatment and 1-year mortality after acute myocardial infarction: insights from the TRIUMPH registry (Translational Research Investigating Underlying Disparities in Acute Myocardial Infarction Patients' Health Status). Circulation.

[12] Helvik AS, Corazzini K, Selbæk G (2016). Health-related quality of life in older depressed psychogeriatric patients: one year follow-up. BMC Geriatr.

[13] Daniel M, Agewall S, Berglund F (2018). Prevalence of anxiety and depression symptoms in patients with myocardial infarction with non-obstructive coronary arteries. Am J Med.

[14] Lichtman JH, Bigger JT, Blumenthal JA (2008). Depression and coronary heart disease: recommendations for screening, referral, and treatment: a science advisory From the American Heart Association Prevention Committee of the Council on Cardiovascular Nursing, Council on Clinical Cardiology, Council on Epidemiology and Prevention, and Interdisciplinary Council on Quality of Care and Outcomes Research: Endorsed by the American Psychiatric Association. Circulation.

[15] Feng L, Li L, Liu W, Yang J, Wang Q, Shi L, Luo M (2019). Prevalence of depression in myocardial infarction: a PRISMA-compliant meta-analysis. Medicine.

[16] Saeed H, Khan F, Mohsin SF, Qizilbash FH, Fraz TR, Jawed Q, Lashari N (2018). Pattern of depression among patients of myocardial infarction in Karachi, Pakistan: a cross-sectional study. Cureus.

[17] Lin Y, Bai W, Liu HH (2023). Prevalence, correlates, and network analysis of depression and its association with quality of life in survivors with myocardial infarction during the COVID-19 pandemic. J Affect Disord.

[18] Džubur A, Lisica D, Hodžić E (2022). Relationship between depression and quality of life after myocardial infarction. Medicinski Glasnik.

[19] Saquib J, AlRomaih NA, Al-Mutairi HM (2018). Correlates of memory loss and depression among myocardial infarction patients in Al-Qassim, Saudi Arabia. J Saudi Heart Assoc.

[20] Wilkowska A, Rynkiewicz A, Wdowczyk J, Landowski J, Cubała WJ (2019). Heart rate variability and incidence of depression during the first six months following first myocardial infarction. Neuropsychiatr Dis Treat.

[21] Iozzia G, De Miranda Azevedo R, Van Der Harst P, Rosmalen JGM, De Jonge P, Roest AM (2020). Association of recognized and unrecognized myocardial infarction with depressive and anxiety disorders in 125,988 individuals: a report of the Lifelines Cohort study. Psychosom Med.

[22] Turen S, Turen S (2023). Gender differences in early complications after STEMI and their associations with anxiety and depression. Eur Rev Med Pharmacol Sci.

[23] Wilkowska A, Rynkiewicz A, Wdowczyk J, Landowski J (2017). Morning and afternoon serum cortisol level in patients with post-myocardial infarction depression. Cardiol J.

[24] Parikh NS, Salehi Omran S, Kamel H, Elkind MS, Willey J (2020). Symptoms of depression and active smoking among survivors of stroke and myocardial infarction: an NHANES analysis. Prev Med.

[25] Worcester MU, Goble AJ, Elliott PC (2018). Mild depression predicts long-term mortality after acute myocardial infarction: a 25-year follow-up. Heart Lung Circ.

[26] Myers V, Gerber Y, Benyamini Y, Goldbourt U, Drory Y (2011). Post-myocardial infarction depression: Increased hospital admissions and reduced adoption of secondary prevention measures — a longitudinal study. J Psychosom Res.

[27] Parakh K, Thombs BD, Fauerbach JA, Bush DE, Ziegelstein RC (2008). Effect of depression on late (8 years) mortality after myocardial infarction. Am J Cardiol.

[28] Wei M, Hornung CA (1994). Depression and survival following myocardial infarction. JAMA.

[29] Thombs BD, Bass EB, Ford DE (2006). Prevalence of depression in survivors of acute myocardial infarction. J Gen Intern Med.

[30] Bahall M (2019). Prevalence and associations of depression among patients with cardiac diseases in a public health institute in Trinidad and Tobago. BMC Psychiatry.

[31] Rothenbacher D, Hahmann H, Wüsten B, Koenig W, Brenner H (2007). Symptoms of anxiety and depression in patients with stable coronary heart disease: prognostic value and consideration of pathogenetic links. Eur J Cardiovasc Prev Rehab.

[32] Larsen KK, Vestergaard M, Søndergaard J, Christensen B (2012). Screening for depression in patients with myocardial infarction by general practitioners. Eur J Prevent Cardiol.

[33] de Jonge P, Spijkerman TA, van den Brink RH, Ormel J (2006). Depression after myocardial infarction is a risk factor for declining health related quality of life and increased disability and cardiac complaints at 12 months. Heart.

[34] Murphy B, Le Grande M, Alvarenga M, Worcester M, Jackson A (2019). Anxiety and depression after a cardiac event: prevalence and predictors. Front Psychol.

[35] Whooley MA, Wong JM (2013). Depression and cardiovascular disorders. Ann Rev Clin Psychol.

